# Osseous choristoma: Report of a case on the palate and a literature review

**DOI:** 10.1002/ccr3.8355

**Published:** 2023-12-21

**Authors:** Nafise Shamloo, Kiarash Modanloo, Armin Khaleghi

**Affiliations:** ^1^ Department of Oral and Maxillofacial Pathology, School of Dentistry Shahid Beheshti University of Medical Sciences Tehran Iran; ^2^ Dental Research Center, Research Institute of Dental Sciences, School of Dentistry Shahid Beheshti University of Medical Sciences Tehran Iran

**Keywords:** choristoma, oral mucosa, osseous, palate

## Abstract

**Key Clinical Message:**

Osseous choristoma is a rare entity, mainly found on the posterior tongue. It is described as a nodular or exophytic lesion with firm to hard consistency. Clinicians should consider osseous choristoma when confronting lesions with the same features.

**Abstract:**

Osseous choristoma is an unusual growth of ectopic bone in the soft tissue. This lesion is extremely rare, with a few cases reported in the literature, and they typically appear in the head and neck region, particularly the posterior tongue. The current report presents a case of osseous choristoma in the palate of a 51‐year‐old female. The patient had slight discomfort, which was resolved after surgical excision of the lesion, and no recurrence was observed. This research presents an instance of osseous choristoma in a less common location and concurrently acts as a review of this rare condition.

## Graphical abstract

Microscopic sections of the lesion revealed hard tissue composed of osseous tissue surrounded by cartilage, which was covered with parakeratnized squamous epithelium.

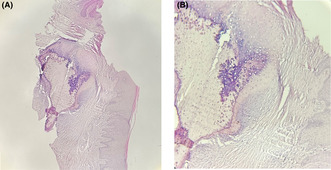

## 1 INTRODUCTION

Choristoma is considered an abnormal but non‐neoplastic growth of histologically normal tissue in an ectopic body location.^1^, ^2^ Choristoma can originate from many different tissues, including bone, cartilage, salivary glands, or muscle, and they are named after the tissue of origin.^3^, ^4^, ^5^, ^6^, ^7^, ^8^ Osseous choristomas are typically composed of thin bones with harvester‐like structures.^2^ The cause of their formation is believed to be either a developmental deformity or a reactive mechanism.^9^ These lesions tend to manifest more frequently in the head and neck region, particularly at the posterior of the tongue. Moreover, they are found mostly in the third and fourth decades of life and are more common in females.^2^, ^9^

Nevertheless, oral choristomas, specifically the osseous variant, are extremely rare findings, with very few documented cases in the existing literature. It is, therefore, of paramount importance to conduct a study on this topic. In addition to providing a literature review on features of this lesion reported in the literature, this case report aims to present a unique case in a less frequently observed location. This endeavor seeks to expand the knowledge on this condition.

## 2 CASE REPORT

A 51‐year‐old healthy female was referred to the Oral and Maxillofacial Surgery Department. The patient presented with mild oral discomfort but reported no pain. On clinical examination, a 10‐mm pink swelling was observed in the posterior midline of the hard palate. The swelling had a smooth surface and firm consistency, appeared pedunculated, and was covered with normal mucosa. The patient had no history of infection or trauma.

Reactive, salivary gland, and mesenchymal lesions are possibilities for an exophytic lesion in the oral cavity. Among these, reactive lesions are more prevalent. Irritation fibroma was considered as the first diagnosis due to its similar appearance, and also, the common occurrence in the oral cavity and palatal area. Peripheral ossifying fibroma was ruled out based on location. Additionally, palatal torus and other bony lesions were ruled out due to the firm consistency of the reported lesion. A radiographic image was not essential at the time due to this diagnosis. Under local anesthesia, the lesion was excised completely. Grossly, the lesion was measured 7 × 3 × 2 mm.

**FIGURE 1:**
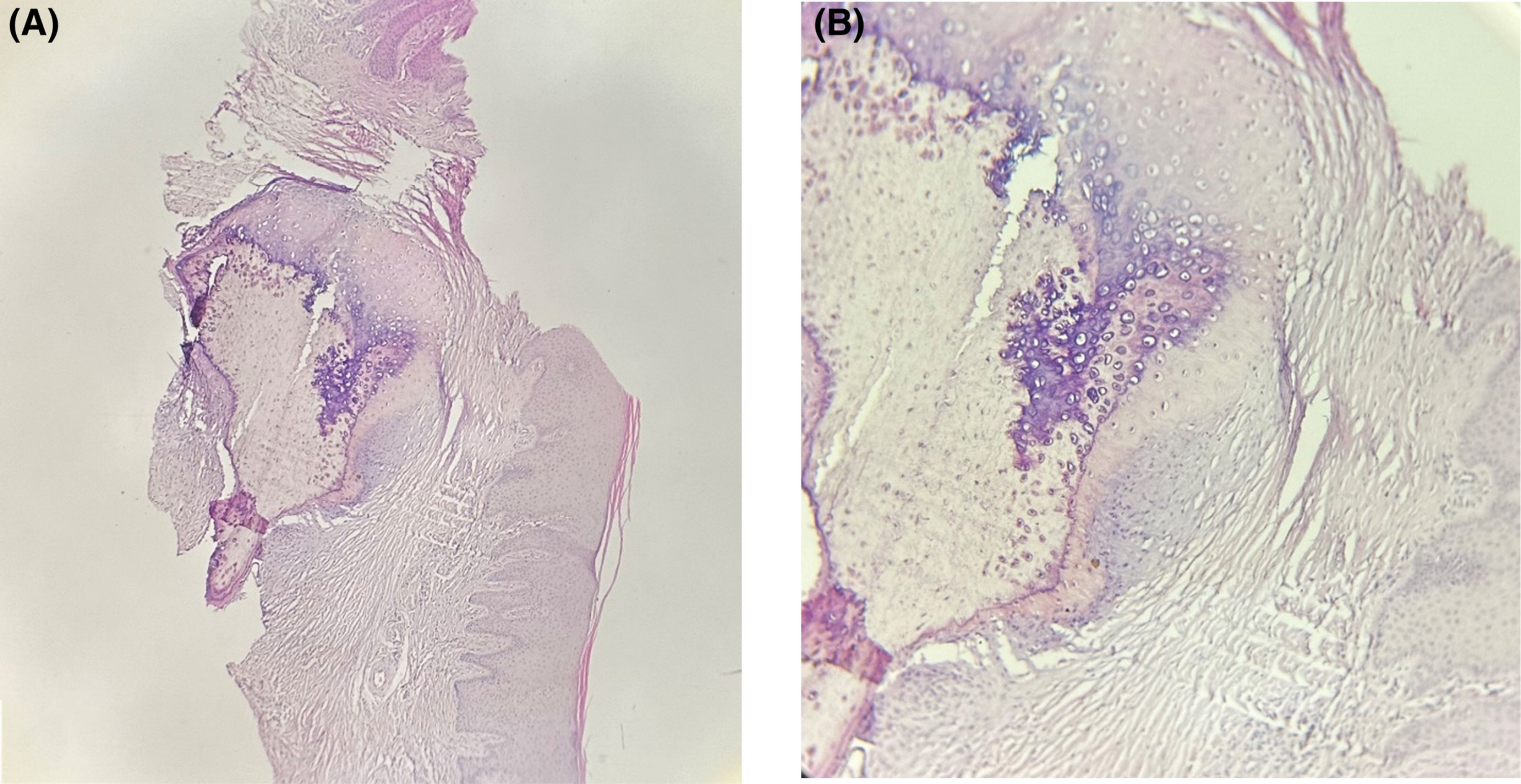
Microscopic views of osseous choristoma, (A) 4× magnification, (B) 10× magnification.

The histopathologic sections revealed hard tissue composed of osseous tissue surrounded by cartilage (Figure 1). The lesion was covered by parakeratinized squamous epithelium. Therefore, based on the microscopic features, the diagnosis of osseous choristoma was made.

The patient's discomfort significantly improved post‐surgery; a three‐month follow‐up showed no signs of recurrence.

## 3 DISCUSSION

The term “choristoma” was initially introduced in 1913 in a report by Monserrat.^10^ Subsequently, numerous investigations have been conducted on lesions exhibiting comparable features. The majority of these studies have focused on the tongue. However, less frequent locations within the head and neck region have also been documented, as similar to the case with our research. Consequently, we have undertaken a search on this subject matter. Previous literature was evaluated using “Osseous OR Bony” AND “Choristoma” queries in PubMed and Google Scholar search engines. A total of 139 cases of osseous choristoma were found. Among these reported cases, 107 (77%) were found in the tongue, which makes it the most common site for osseous choristoma of the head and neck. Following the tongue, 13 cases (9.4%) were reported in the buccal mucosa. Osseous choristoma of the palate was mentioned previously in three separate reports, which makes our report the fourth one in the list. Other studies reported this lesion in less common sites, including gingiva,^11^, ^12^, ^13^, ^14^, ^15^ alveolar mucosa,^16^ submental region,^17^, ^18^ submandibular region,^19^ masseter,^20^ medial aspect of the lateral pterygoid,^21^ lips,^22^, ^23^ and tonsils.^24^

The mean age for the reported cases were 31.97 ± 18.04, ranging between 1 month to 89 years old. The youngest patient ever diagnosed with osseous choristoma was a 1‐month‐old infant girl. In this case, the lesion was accompanied by Tessier no.7 or transverse facial cleft.^25^ Moreover, osseous choristoma tends to appear more in females and the mean age of the female patients was relatively lower than males.

Regarding clinical features, 58 cases (41.7%) were pedunculated, 17 cases (12.2%) were sessile, and 30 cases (21.6%) were described as nodular masses. The consistency of the lesions was stated as firm in 23 cases, hard in 18 cases, and bony hard in 14 cases. These specific features were not consistently documented in previous studies. In terms of lesion size, the largest osseous choristoma ever reported was 10 cm, located on the tongue.^26^ However, most of them were between 0.5 to 2 cm. Osseous choristomas on the lips and the palate had an average size of less than 1 cm, which is relatively smaller than lesions in other sites. The size discrepancy may be attributed to the limited number of reported cases in these locations or to the fact that lesions on the lips and palate are more visually conspicuous, leading to earlier diagnoses. Table 1 compares osseous choristomas location‐wise.

**TABLE 1:** Locations of osseous choristoma.

Location	Frequency *N* (%)	Gender (*N*)			Recurrent cases (*N*)
		Male	Female	Unknown	
Tongue	107	28	73	6	–
Buccal mucosa	13	4	8	1	2
Gingiva	5	2	3	–	–
Palate	3	2	1	–	–
Lips	2	1	1	0	–
Submental	2	0	2	0	–
Tonsil	2	1	1	–	1
Alveolar mucosa	1	1	0	–	–
Lateral pterygoid	1	0	1	–	–
Masseter	1	1	0	–	–
Retromolar pad	1	1	0	–	–
Submandibular	1	1	0	–	–
Total	139	42	90	7	3

Abbreviations: *N*, number; SD, standard deviation.

Most patients affected by osseous choristoma were asymptomatic or no symptoms were reported (51.7%). However, among symptomatic patients, various manifestations were observed. The most common symptom was the feeling of a lump or a swelling (23.7%), followed by dysphagia (8.6%), pain (5.7%), gag feeling (5%), globus pharyngeus (3.6%), hoarseness (2.9%), and odynophagia (1.4%). Also, airway distress, snoring, bleeding, infection, and ulceration were reported in one case each.

In most of the reports, the duration of the lesion's existence was asked from the patient. In 34 cases (24.5%), the lesions were found upon oral examination and patients were not aware of the lesion. However, 16 patients (11.5%) were aware of the lesion for less than 1 year, 33 (23.7%) between 1 to 10 years, and 5 (3.6%) for more than 10 years. Four patients (2.9%) were alert of the lesion since birth or precisely since they had remembered.

Some of the studies reported a history of the patients, whether medical, habitual, or any trauma that could have contributed to lesion formation. No pattern was observed in the history of patients. Three patients mentioned smoking, all of whom had tongue lesions. Only one of the patients with a lesion on the upper lip, reported trauma to the site before the lesion formation.^23^ Also, gastroesophageal reflux disease (GERD) was mentioned in three cases which could have contributed as a traumatic factor in the lesion growth.

Treatment of osseous choristoma is surgical removal. Out of the 139 reported cases in the literature, only three of them were recurrent. Two of them were on the buccal mucosa and one was on the masseter muscle. Recurrency could be explained if we suppose that these lesions are of traumatic origin and the recurrent lesion was the result of surgical trauma.^27^ In addition, it could have been a similar lesion that was found later due to a slower increase in size or delayed ossification. Further reports are needed for an explanation of recurrence.

Numerous osseous lesions can manifest on the palate, and a broad spectrum of differential diagnoses should be considered. These may include palatal torus, ossifying myositis, ossifying fasciitis, peripheral ossifying fibroma, progressive ossifying fibrodysplasia, intraosseous osteosarcoma, osteolipoma, phlebolith, and sialolith.^27^ Comparison between palatal osseous choristomas is illustrated in Table 2. Our study serves as the fourth report of osseous choristoma on the palate.

**TABLE 2:** Palatal osseous choristoma.

Study	Year	Gender	Age	Size (cm)	Clinical presentation	Symptoms
Feenstra et al.^28^	1977	Male	14	NR	NR	NR
Wada et al.^27^	2010	Female	16	0.9	Nodular/hard	None
Sasaki et al.^29^	2016	Male	37	0.7	Pedunculated/hard	None
Present case	2023	Female	51	1.0	Pedunculated/firm	Discomfort

Abbreviation: NR, not reported.

Several studies have been conducted to unravel the etiology of osseous choristomas. Previous research suggested a congenital basis for these lesions.^25^, ^30^ The tongue is formed by the fusion of two parts: the anterior two‐thirds of the tongue is derived from the first branchial arch, while the posterior third is from the third branchial arch. These branchial arches also give rise to normal osseous structures such as the hyoid bone, malleus, incus, and styloid process, through a process called endochondral ossification.^31^ The developmental theory posits that oral choristoma arises from the entrapment of branchial arches I, II, and III derivatives in the facial region. As most lesions are located at the midline, this explanation appears plausible.^30^ However, this theory falls short of providing a complete understanding of the full range of lingual or other intraoral osseous choristomas and the higher incidence of such lesions in females.^23^, ^32^ Environmental factors have also been proposed to contribute to intraoral osseous choristoma development, challenging the congenital viewpoint.^33^, ^34^, ^35^ Nonetheless, the presence of well‐developed lamellar bone is not consistent with chronic irritation.^29^ Conducting more research in this field could yield valuable insights and aid in establishing a correlation between environmental factors and the formation of intraoral osseous choristomas.

While the role of environmental factors in the development of intraoral osseous choristomas should not be disregarded, a multifactorial etiology is probably involved. Further research is necessary to establish the precise causality of these lesions.

## AUTHOR CONTRIBUTIONS

**Nafise Shamloo:** Investigation; methodology; supervision; writing – review and editing. **Kiarash Modanloo:** Data curation; investigation; resources; validation; writing – original draft. **Armin Khaleghi:** Conceptualization; formal analysis; investigation; project administration; writing – original draft.

## FUNDING INFORMATION

None.

## CONFLICT OF INTEREST STATEMENT

The authors have no conflicts of interest to declare.

## CONSENT

Written informed consent was obtained from the patient to publish this report in accordance with journal's patient consent policy.

## Data Availability

The data that support the findings of this study are available on request from the corresponding author. The data are not publicly available due to privacy or ethical restrictions.
